# The effect of local anaesthetic wound infiltration on chronic pain after lower limb joint replacement: A protocol for a double-blind randomised controlled trial

**DOI:** 10.1186/1471-2474-12-53

**Published:** 2011-02-26

**Authors:** Vikki Wylde, Rachael Gooberman-Hill, Jeremy Horwood, Andrew Beswick, Sian Noble, Sara Brookes, Alison J Smith, Mark Pyke, Paul Dieppe, Ashley W Blom

**Affiliations:** 1 Musculoskeletal Research Unit, School of Clinical Sciences, University of Bristol, Avon Orthopaedic Centre, Southmead Hospital, Bristol, BS10 5NB, UK; 2 School of Social and Community Medicine, University of Bristol, Canynge Hall, 39 Whatley Road, Bristol, BS8 2PS, UK; 3 North Bristol NHS Trust, Avon Orthopaedic Centre, Southmead Hospital, Bristol, BS10 5NB, UK; 4 Peninsula Medical School, Universities of Exeter and Plymouth, Plymouth, PL6 8BU, UK

## Abstract

**Background:**

For the majority of patients with osteoarthritis (OA), joint replacement is a successful intervention for relieving chronic joint pain. However, between 10-30% of patients continue to experience chronic pain after joint replacement. Evidence suggests that a risk factor for chronic pain after joint replacement is the severity of acute post-operative pain. The aim of this randomised controlled trial (RCT) is to determine if intra-operative local anaesthethic wound infiltration additional to a standard anaethesia regimen can reduce the severity of joint pain at 12-months after total knee replacement (TKR) and total hip replacement (THR) for OA.

**Methods:**

300 TKR patients and 300 THR patients are being recruited into this single-centre double-blind RCT. Participants are recruited before surgery and randomised to either the standard care group or the intervention group. Participants and outcome assessors are blind to treatment allocation throughout the study. The intervention consists of an intra-operative local anaesthetic wound infiltration, consisting of 60 mls of 0.25% bupivacaine with 1 in 200,000 adrenaline. Participants are assessed on the first 5 days post-operative, and then at 3-months, 6-months and 12-months. The primary outcome is the WOMAC Pain Scale, a validated measure of joint pain at 12-months. Secondary outcomes include pain severity during the in-patient stay, post-operative nausea and vomiting, satisfaction with pain relief, length of hospital stay, joint pain and disability, pain sensitivity, complications and cost-effectiveness. A nested qualitative study within the RCT will examine the acceptability and feasibility of the intervention for both patients and healthcare professionals.

**Discussion:**

Large-scale RCTs assessing the effectiveness of a surgical intervention are uncommon, particulary in orthopaedics. The results from this trial will inform evidence-based recommendations for both short-term and long-term pain management after lower limb joint replacement. If a local anaesthetic wound infiltration is found to be an effective and cost-effective intervention, implementation into clinical practice could improve long-term pain outcomes for patients undergoing lower limb joint replacement.

**Trial registration:**

Current Controlled Trials ISRCTN96095682

## Background

Joint replacement is one of the most common elective surgical procedures, with over 150,000 total hip replacements (THR) and total knee replacements (TKR) performed annually in England and Wales in the National Health Service (NHS)[1]. Although chronic pain is the main indication for joint replacement, it is also a common occurrence after surgery. Studies have estimated the incidence of chronic pain after joint replacement to be in the region of 10-30% [2-4]. Chronic pain after surgery is defined by the International Association for the Study of Pain (IASP) as pain that develops after a surgical intervention and is of at least 3-months duration[5]. Chronic pain is a widely-observed phenomenon after many surgical procedures including breast surgery[6], inguinal hernia repair[7] and thoracic surgery [8]. Research evidence suggests that an important risk factor for chronic pain after surgery is the severity of acute post-operative pain[9,10]. Therefore, in addition to causing unnecessary distress and discomfort during the post-operative period, poorly managed post-operative pain can have negative consequences for long-term surgical outcomes. Adequate management of post-operative pain still poses a significant challenge to healthcare, as demonstrated by a review of the literature which concluded that 30% of patients experience moderate or severe pain after surgery [11]. In a recent study at the Avon Orthopaedic Centre, 58% of TKR patients and 47% of THR patients reported moderate or severe pain on the first post-operative day[12]. Therefore, there is a need for trials of interventions targeting acute post-operative pain with the ultimate aim of improving long-term outcomes after joint replacement.

In the context of joint replacement, post-operative pain management needs to provide effective pain relief, while allowing for early mobilisation and rehabilitation[13]. Although many of the traditional methods of achieving peri-operative pain relief can hinder early mobilisation[14], a review of the use of multimodal analgesia in different conditions found that a combination of systemic opioids, nonsteroidal anti-inflammatory drugs, and epidural analgesics or wound infiltration can provide effective pain control and improve post-operative recovery[15]. In joint replacement, there is evidence that multimodal analgesia can give good pain relief with minimal side effects [16,17]. A systematic review of a wide range of surgical procedures concluded that the use of local anaesthetic wound infiltration as part of a multimodal approach reduced post-operative pain, opioid use, nausea and vomiting, improved patient satisfaction and reduced the length of hospital stay[18]. Fifteen randomised controlled trials (RCT) in joint replacement identified up to August 2010 are described in Table 1[19-33]. The results from these studies found that short-term outcomes after joint replacement using multimodal analgesia were broadly favourable. However, with only one small study reporting outcomes after six months, there is little evidence on longer-term pain outcome.

**Table 1:** Randomised controlled trials of local anaesthetic wound infiltration in hip or knee joint replacement

Author	Study design Patients Longest follow up	Common treatment		Results
			
		Intervention	Control	
Bianconi 2003[20] Italy	RCT TJR N = 37 72 hours	*Spinal anaesthsia with bipuvicaine and a loading dose of intravenous morphine 10 mg. Intravenous infusion of normal saline at the same rate for 24 hours*		VAS pain at rest and on mobilisation significantly reduced in the intervention group up to 72 hours. Use of diclofenac and tramadol lower in the intervention group, and length of hospital stay reduced. Patient satisfaction greater in the intervention group.
			
		Wound infiltration with ropivacaine solution followed by wound perfusion for 55 hours	Baseline intra-venous infusion of morphine plus ketorolac for 24 hours	

Toftdahl 2007 [21] Sweden	RCT TKR N = 80 24 hours	*Spinal anaesthesia and controlled release oxycodon*		Intervention group had a quicker recovery, lower pain scores, and lower use of opioids. There was no difference in side effects or length of hospital stay.
			
		Peri- and intraarticular infiltration and injection	Continuous femoral nerve block	

Andersen 2007 [22] Denmark	RCT THR N = 80 Until discharge	*Spinal anaesthesia*		Pain reduced in intervention group. Narcotic consumption was reduced and the length of hospital stay was shorter.
			
		Wound infiltration and intraarticular injection of local anaesthetic	Epidural infusion	

Andersen 2007 [23] Denmark	RCT THR N = 40 6 weeks	*Spinal anaesthesia*		Patients who received the active wound infiltration had less pain for up to 2 weeks. They reached an earlier and lower pain minimum during the first days post-operatively, had lower use of analgesia up to day 4 post-operatively, and were more satisfied. Use of analgesic solution resulted in less joint stiffness and better function 1 week postoperatively. No significant benefit at 6 weeks.
			
		Wound infiltration with ropivacaine, ketorolac, and adrenaline at the end of surgery and through an intraarticular catheter 24 hours post-operatively	Saline infiltration	

Vendittoli 2006 [33] Canada	RCT TKR N = 42 24 hours	*Spinal anaesthesia and patient controlled morphine*		Morphine use and the incidence of nausea was significantly lower in the intervention group.
			
		Perioperative infiltration of local anesthetic		

Busch 2006 [25] Canada	RCT TKR N = 64 6 weeks	*Spinal anaesthesia and patient controlled morphine*		Patient-controlled analgesia use was reduced in the 24 hours after surgery in the intervention group. VAS pain scores also improved in the intervention group after the operation. No significant benefit at 6 weeks.
			
		Locally injected anaesthetic		

Andersen 2008 [29] Denmark	Individual patient knees randomised to intervention or control. TKR (bilateral) N = 12 48 hours	*Spinal anaesthesia and patient controlled morphine*		Pain at rest and during movement significantly reduced for up to 32 hrs in the intervention group.
			
		Infiltration with ropivacaine and epinephrine. Further intra-articular injections through catheter over 24 hours.	Similar procedures with saline.	

Essving 2009 [32] Sweden	RCT Unicompartmental knee arthroplasty N = 40 6 months	*General anaesthesia and patient controlled *Essving *morphine*		Hospital stay shorter in intervention group. Post-operative pain lower at rest during 27 hr in-hospital follow up. Morphine consumption lower for the first 48 hr in intervention group. Also lower frequency of nausea, pruritus, and sedation. No benefit in Oxford knee score and EQ-5 D in intervention group at 3 or 6 months.
			
		Periarticular infiltration of ropivacaine, ketorolac and epinephrine. Further injection through catheter at 21 hrs.	Similar volume of saline injected via catheter	

Fu 2009 [19] China	RCT TKR N = 80 15 days (pain)	*Spinal anaesthesia and patient controlled morphine*		Significant reduction in morphine consumption. VAS pain at rest and activity improved up to 36 hours. No difference in VAS pain from 48 hours.
			
		Intraoperative intra-articular injection and infiltration of morphine, bupivacaine and betamethasone	Similar procedure with saline	

Parvataneni 2007 [30] USA	RCT TKR N = 60 3 months	*Spinal anaesthesia with or without femoral nerve block*		Non-significant reduction in VAS pain and greater satisfaction in intervention group on day 1 but later VAS pain and satisfaction were similar. Narcotic consumption was reduced in the intervention group. The intervention was associated with quicker functional recovery but length of hospital stay was similar between the groups.
			
		Local periarticular injection of bupivacaine, morphine sulfate, epinephrine, methylprednisolone acetate, cefuroxime	Patient controlled analgesia (ketoralac and, if ineffective, morphine)	

Parvataneni 2007 [30] USA	RCT THR N = 71 3 months	*Spinal anaesthesia with or without femoral nerve block*		VAS pain reduced in intervention group at 3 days but similar at 6 weeks and 3 months. Satisfaction greater in hospital in intervention group but similar at 6 weeks and 3 months. Lower narcotic use in intervention group. Functional recovery was quicker and the hospital stay shorter in the intervention group.
			
		Local periarticular injection of bupivacaine, morphine sulfate, epinephrine, methylprednisolone acetate, cefuroxime	Patient controlled analgesia (ketoralac and, if ineffective, morphine)	

Zhang 2007[31] China	RCT TKR N = 60 72 hours	*Patient controlled analgesia*		VAS pain at rest and on activity reduced in intervention group until 24 hours. No difference at 36-72 hours. Reduced post-operative tramadol use in intervention group. Range of motion improved in intervention group at 72 hours.
			
		Intraoperative periarticular injection of bupivacaine, epinephrine and morphine	No drug infiltration	

Gomez- Cardero 2010 [26] Spain	RCT TKR N = 50 1 month	*Spinal anaesthesia *Cardero		Pain intensity and opiod use in the first 3 days post-operative were reduced in the intervention group. Length of hospital stay was lower in the intervention group. There was no difference in pain intensity at 1- month post-operative.
			
		Continous intraarticular infusion with ropivacaine	Saline infusion	

Essving 2010 [27] Sweden	RCT TKR N = 48 3 months	*General anaesthesia and patient controlled morphine*		Morphine consumption and pain intensity on movement was reduced in the intervention group in the first 48 hours post-operative. Patient satisfaction was higher in the intervention group. Length of stay was not affected and no significant benefit was evident at 3-months post-operative.
			
		Intraarticular injection of ropivacaine, ketorolac and epinephrine during surgery and through a catheter at 21 hours post-operatively	No injection during surgery and saline injection at 21 hours post-operatively	

Chen 2010[28] Taiwan	RCT THR N = 92 Discharge	*General anaesthesia*		Intervention group had a longer mean time to first narcotic rescue, but there was no difference in pain relief, narcotic use or length of stay.
			
		Continous intra-articular infusion of bupivacaine via a infusion pump for 48 hours	Continous intra-articular infusion of saline via a infusion pump for 48 hours	

RCT = randomised controlled trial, TKR = total knee replacement, THR = total hip replacement, TJR = total joint replacement

The existing literature provides evidence that the use of local anaesthetic wound infiltration as part of a multimodal anaesthetic regimen reduces short-term post-operative pain after joint replacement. However, it is not known if the reduction of this short-term post-operative pain reduces the severity of chronic pain. The aim of this RCT is to determine if using intra-operative local anaesthetic wound infiltration significantly reduces the severity of joint pain at 12-months after primary TKR or THR.

## Methods

The Arthroplasty Pain Experience (APEX) study is a single-centre double-blind RCT that is currently being conducted at the Avon Orthopaedic Centre, one of the largest orthopaedic centres in the UK. The study has been approved by Southampton and South West Hampshire Research Ethics Committee (B) (09/H0504/94) and all participants provide informed, written consent. The trial has been registered with EudraCT (2009-013817-93) and Current Controlled Trials (ISRCTN96095682). The trial is also registered as a Clinical Trial of a Investigational Medicinal Produce with the Medicine Healthcare and Regulary Authority (18524/0215/001-0001).

### Study duration

Recruitment into the trial began in November 2009 and 12-month follow-up for all participants is anticipated to be complete by November 2012.

### Participant recruitment

Potential participants are screened and recruited by research nurses from pre-operative assessment clinics at the Avon Orthopaedic Centre. Inclusion criteria include being listed for a primary unilateral TKR or THR for osteoarthritis (OA) and being willing and able to provide fully-informed consent. Exclusion criteria include i.) any medical co-morbidity that precludes spinal anaesthesia, regional blocks or the use of strong analgesics post-operatively ii.) severe dementia or psychiatric illness such that they are unable to complete the questionnaires or provide informed consent iii.) listed for simultaneous bilateral joint replacement iv.) having been in the APEX trial for a previous joint replacement v.) being unable to understand English as not all the questionnaires have been translated and validated into other languages. In order to explore generalisability (the patients enrolled in the study being representative of those undergoing joint replacement) anonymised data about age and gender is recorded for all eligible patients.

### Randomisation

Before participants undergo surgery, they are randomised to either the standard care group or the intervention group. Randomisation is conducted by means of a computer-generated code, administered centrally and communicated via the internet (through the Bristol Randomised Trials Collaboration). Randomisation is stratified by operation site (TKR/THR) and minimised by baseline joint pain severity and surgical approach. The operating surgeon and anaesthetist are blind to the results of randomisation until the beginning of the operation, when they are informed of treatment allocation by a member of the research team who is not involved in outcomes assessment. The study participants, research nurses involved in recruitment and assessment, and clinical staff involved in the care of study participants are blind to treatment allocation throughout the study.

### Intervention

#### Hip replacement participants

##### Standard care group

The standard anaesthetic care for THR participants is a spinal anaesthetic with 3 mls of 0.5% plain bupivacaine placed at the L3,4 or L4,5 interspace. Intra-operatively, the patient is either awake, sedated or under a light general anaesthetic, depending on patient and anaesthetic factors. If there is intra-operative discomfort then rescue analgesia in the form of intravenous fentanyl is titrated to effect. All participants are given 1 g of intravenous paracetamol 30 minutes before the end of the operation. In the recovery area immediately post-operative, participants receive (if no contra-indications are present) 400 mg of ibuprofen administered orally. A patient controlled analgesia (PCA) device is started containing morphine 1 mg/ml, a 1 mg bolus dose and a 5 minute lock-out. If on awakening the patient is in pain with a rating of more than 50 mm on a 100 mm pain visual analogue scale (VAS), a morphine bolus up to 0.2 mg/kg can be administered as rescue analgesia.

Each day during their hospital stay, participants receive a visit from a pain specialist nurse. Post-operative analgesia consists of oral or intravenous paracetamol every 6 hours and, if no contraindications are present, oral ibuprofen 400 mg every 8 hours. When the PCA is no longer needed, oral codeine phosphate 30-60 mg every 6 hours, tramadol 50-100 mg every 6 hours and oramorph 10-20 mg are prescribed as rescue analgesia.

##### Intervention group

The intervention group receives the same anaesthetic and analgesic regime as the standard care group, plus an intra-operative local anaesthetic wound infiltration. The local anaesthetic mixture consists of 60 mls of 0.25% bupivacaine with 1 in 200,000 adrenaline. If the patient is below 60 kg or particularly frail, the volume of injectate is reduced to 50 mls or lower if necessary. The surgeon injects the anaesthetic mixture into the joint capsule and short external rotators, fascia, fat and subcutaneous tissue.

#### Knee replacement participants

##### Standard care group

The standard anaesthetic care for TKR participants is a femoral nerve block and a spinal or general anaesthetic, depending on patient factors. The femoral nerve blockade is achieved by an injection of 20 mls of 0.25% bupivacaine sited with the use of a nerve stimulator and/or ultrasound guidance. Intra-operative analgesia is provided by titration of intravenous fentanyl initially and morphine if deemed necessary in order to achieve haemodynamic stability and a smooth emergence. All participants are given 1 g of intravenous paracetamol 30 minutes before the end of the operation. The post-operative pain management regime for TKR participants is the same as that described for THR participants.

##### Intervention group

The intervention group receive the same anaesthetic and analgesic regime as the standard care group, plus an intra-operative local anaesthetic wound infiltration. The local anaesthetic mixture is the same as described for THR participants. The surgeon injects the anaesthetic mixture into the posterior capsule, medial and lateral capsule, fascia and muscle, and subcutaneous tissues.

### Assessment times

Participants are followed-up for 12-months after surgery. Assessments are conducted pre-operatively, daily during the hopsital stay, and then at 3-months, 6-months and 12-months post-operative. Outcomes are assessed using self-report questionnaires, joint examinations, analysis of x-rays, pressure algometry and extraction of data from hospital records.

### Primary outcome measure

The primary outcome is the 12-month Western Ontario and McMaster Universities Index of Osteoarthritis (WOMAC) Pain Scale[34]. The WOMAC Pain score is a widely-used and validated questionnaire to assess the severity of joint pain in OA patients[35]. The severity of joint pain is assessed when performing 5 different activities: walking, using stairs, sitting or lying, standing upright and in bed. Response options for each item are on a 5-point ordered response scale, ranging from none to extreme, which are summed and then transformed into a 0-100 scale (extreme pain-no pain).

### Secondary outcome measures

#### Pain severity during the in-patient stay

On the day of surgery, participants are asked to verbally rate their pain severity on a 0-10 scale when they wake up in the recovery room and when they leave recovery, and then on a 4-hourly basis when they are transfeered to a ward. From the day after surgery to discharge (or up to post-operative day 5), participants complete a 100 mm VAS for pain severity at rest and 100 mm VAS for pain severity on movement in the morning, afternoon and evening.

#### Satisfaction with in-patient pain relief

At the end of each day, participants complete a 100 mm VAS to indicate their satisfaction with their pain relief.

#### Nausea and vomiting during the in-patient stay

Participants are asked whether they have experienced any vomiting or nausea (yes/no variable) each day. If participants have experienced nausea, they then indicate how distressing it was on a 100 mm VAS (not at all distressing to very distressing).

#### Length of hospital stay

This is calculated from participants admission and discharge dates.

#### Joint pain and disability

The WOMAC[34] and the Intermittent and Constant Osteoarthritis Pain (ICOAP)[36] are completed pre-operatively, and then at 3-months, 6-months and 12-months post-operatively. To assess whether the intervention reduces the incidence of chronic neuropathic pain, participants complete the PainDETECTquestionnaire[37] at 12-months post-operative.

#### Pressure algometry

Pressure pain thresholds, which are a measure of pain sensitivity, are measured on the volar forearm before surgery, on discharge from hospital, and 12-month post-operative using a pressure algometer (Somedic, Sweden).

#### Medical and surgical complications

This data is recorded from hospital records during the in-patient stay, a telephone call to participants at 3-months post-operative, and a comprehensive joint assessment by a research nurse and an x-ray review at 12-months post-operative. If participants report the occurence of a complication at either 3-months of 12-months post-operative, this is verified by a review of their hospital records.

#### Cost effectiveness

Health service resource data, including staff time and other resources used in the intervention, subsequent inpatient stays, outpatient and community based visits and medication are collected using hospital records and participant self-complete questionnaires. These questionnaires also collect information on social service use, travel, time off work, usual activities, informal care, and include the EQ-5D[38].

### Potential prognostic factors

A number of possible potential prognostic factors are also being recorded, including socio-demographic factors, medical comorbidities, structural joint damage, health-related quality of life and psychosocial factors. The EQ-5D[38] is being used as a measure of health-related quality of life and the following questionnaires are being used to assess psychosocial factors: Hospital Anxiety and Depression Scale [39], Pain Self-Efficacy questionnaire[40], Illness Perceptions Questionnaire-Revised [41] and the Brief COPE [42].

### Nested qualitative study

Within the RCT, there is a nested qualitative study to explore the acceptability and feasibility of the intervention for both patients and healthcare professionals. Interview participants are purposively sampled to include a range of socio-demographic variables so they adequately reflect those of a range of NHS patients undergoing joint replacement and NHS staff involved in joint replacement surgery. Data collection comprises of in-depth qualitative interviews with up to 25 patients at 2-3 weeks post-operative and with up to 20 healthcare professionals involved in the trial. Interviews with patients elicit experiences of surgery and post-operative recovery, acceptability of their treatment, identify information needs regarding anaesthesia and the degree to which they would like to be involved in decision-making about pain management. Interviews with healthcare professionals explore the acceptability and feasibility of the intervention and identify any potential barriers to the implementation of the intervention. The interviews are audio-recorded and fully transcribed, and themes are compared within and between one another using constant comparison techniques[43].

### Sample size calculation

The primary outcome for this trial is the WOMAC Pain score at 12-months post-operative. The trial is powered to allow the THR and TKR groups to be analysed separately. Three hundred patients listed for TKR are being recruited into the study, and 150 patients will be randomised to the intervention arm and 150 to the standard care arm. The same number of patients listed for THR are being recruited and randomised. This sample size will provide 90% power to detect a difference of 0.5 standard deviations on the WOMAC Pain Scale with a two-sided 1% significance level, and allows for a 20% dropout rate. Previous research suggests standard deviations of around 17 mm on the WOMAC Pain Scale before surgery[44]. Hence a difference between the treatment groups of 0.5 standard deviations equates to a difference of approximately 8-9 units on the WOMAC Pain Scale (0-100 scale).

### Statistical analysis

In terms of generalisability, the characteristics of those successfully recruited into the trial will be compared with those who were eligible but declined to participate. A CONSORT diagram [45] will summarise participant flow through the study, documenting invitation and recruitment, receipt of intervention or standard care as allocated, and collection of data. To check for balance between the groups, participants in the intervention and standard care arms will be described separately in terms of demographic, stratification/minimisation variables, and baseline measures. The percentage of participants not providing primary and secondary outcome data at each of the follow-up stages will be reported for each trial arm. Factors associated with missing data (such as demographics and values of primary and important secondary outcome variables at baseline) will be explored.

Analysis will consider TKR and THR separately. The primary analysis will be implemented using a multivariable generalised linear model to investigate between-group differences in mean WOMAC pain score at 12 months follow-up, adjusting for minimisation variables and baseline value of the primary outcome. The type of model will depend on the distribution of the WOMAC pain score data. The primary analysis will be conducted on an intention-to-treat (ITT) basis without imputation. However, sensitivity analyses will inform the interpretation of the primary analysis. These will include an estimate of between-group difference for the primary outcome using a per-protocol analysis. In addition, the effect of missing primary outcome data will be investigated using multiple imputation methods and random effects regression models will be used to investigate any clustering of outcomes by surgeon.

The same general approach will be followed for analysis of secondary outcome measures. For additional exploratory analyses, data will be considered within each treatment group separately and so treated as two cohort studies. Analyses will examine the impact of potential risk factors, specified a priori (see section on potential prognostic factors), on pain severity at 12-months post-operative independent of any treatment effects.

### Health economic analysis

The aim of the economic evaluation is to compare from a societal perspective the costs and effects of the intervention compared to standard care. Resource use in relation to joint replacement is collected from the date of operation until the end of the trial. Health service resource use is valued using the hospital finance department data, as well as routine UK data sources. Patient and informal carer resource use is valued using self-report and routine data sources. The analysis will consider TKR and THR participants separately. The analysis will first estimate the between group differences in mean costs using bootstrapping techniques to obtain bias corrected confidence intervals if the data are skewed. Then, incremental cost effectiveness between the two arms of the trial will be estimated using the WOMAC Pain score and the EQ-5 D to calculate Quality Adjusted Life Years (QALYs) for this population during the trial period. These results will be presented as incremental cost-effectiveness ratios and cost-effectiveness acceptability curves, derived used bootstrapping techniques. These will show the probability of the intervention being cost-effective at a range of 'willingness to pay' threshold levels.

The net monetary benefit statistic, using the difference in costs and the difference in QALYs between the two arms of the trial, will also be calculated for the different values of the societal willingness to pay for a year of life. In addition, because of the number of important secondary outcomes, a cost consequence analysis will be conducted in which the difference in costs and the difference in effects between the two arms of the trial are presented in tabular form. Sensitivity analysis will then be conducted to take account of uncertainty and imprecision in measurements, and will include multiple imputation models for missing values. All these analyses will be on an ITT basis and will adjust for minimisation variables.

## Discussion

Many people experience chronic pain after a range of different surgical procedures[2-4,6-8], and this pain is often accompanied by considerable psychological distress[46]. For patients undergoing joint replacement, chronic pain can be particularly distressing because the main reason for electing to undergo surgery is to gain relief from chronic joint pain. Therefore, for the 10-30%[2-4] of patients who experience severe chronic pain after joint replacement, the surgery has been unsucessful in achieving its primary aim. The number of patients who develop chronic pain after joint replacement will continue to increase as the need for joint replacement increases[47], and therefore it is important that interventions to optimise outcomes after joint replacement are evaluated.

RCTs are the highest level of evidence to assess the effectivness of a surgical intervention. However, there is a paucity of well-designed and sufficiently-powered RCTs of surgical interventions, particulary within orthopaedics. Common problems with the design and reporting of orthopaedic-based RCTs include patients and outcome assessors not being blinded to treatment allocation, use of unvalidated or modified validated outcome measures, no sample size calculation being performed prior to the trial, unclear inclusion and exclusion criteria, and lack of information on the method of statistical analysis [48-51]. The APEX trial overcomes these limitations, as demonstrated by its prospective double-blind randomised design, use of a validated primary outcome measure, sufficient statistical power to detect a clinically meaningful reduction in long-term pain severity, and clearly defined methods of analysis. In addition, the APEX trial benefits from the inclusion of a cost-effectiveness analysis and a nested qualitative study to assess the acceptability and feasibility of trial participation and of the intervention.

The primary aim of the APEX trial is to determine if the addition of an intra-operative local anaesthetic wound infiltration to the standard anaesthetic regime at the Avon Orthopaedic Centre can significantly reduce the severity of joint pain at 12-months after primary THR and TKR. The results from this trial will inform evidence-based recommendations for both short-term and long-term pain management after lower limb joint replacement. If a local wound infiltration is found to be an effective and cost-effective intervention, its implementation into clinical practice could reduce the severity of chronic pain after THR and TKR, and therefore improve long-term pain outcomes for patients undergoing lower limb joint replacement.

## Competing interests

The authors declare that they have no competing interests.

## Authors' contributions

All authors conceived and designed the trial

SN designed the health economic evaluation

SB and AS designed the statistical analysis

RGH and JH designed the nested qualitative study

All authors drafted the manuscript

## Pre-publication history

The pre-publication history for this paper can be accessed here:

http://www.biomedcentral.com/1471-2474/12/53/prepub

## References

[B1] National Joint Registry 7th Annual Report 2010

[B2] Wylde V Dieppe P Hewlett S Learmonth ID Total knee replacement: Is it really an effective procedure for all? Knee 2007 14 6 417 423 10.1016/j.knee.2007.06.001 17596949

[B3] Nikolajsen L Brandsborg B Lucht U Jensen TS Kehlet H Chronic pain following total hip arthroplasty: a nationwide questionnaire study Acta Anaesthesiol Scand 2006 50 4 495 500 10.1111/j.1399-6576.2006.00976.x 16548863

[B4] Wylde V Hewlett S Learmonth I Dieppe P Persistent pain after joint replacement: prevalence, sensory qualities and post-operative determinants Pain 2011 152 3 566 572 10.1016/j.pain.2010.11.023 21239114

[B5] International Association for the Study of Pain Classification of chronic pain. Descriptions of chronic pain syndromes and definitions of pain terms. Prepared by the International Association for the Study of Pain, Subcommittee on Taxonomy Pain Suppl 1986 3 S1 226 3461421

[B6] Caffo O Amichetti M Ferro A Lucenti A Valduga F Galligioni E Pain and quality of life after surgery for breast cancer Breast Cancer Res Treat 2003 80 1 39 48 10.1023/A:1024435101619 12889597

[B7] Poobalan A Bruce J King P Chambers W Krukowski Z Smith W Chronic pain and quality of life following open inguinal hernia repair British Journal of Surgery 2001 88 8 1122 1126 10.1046/j.0007-1323.2001.01828.x 11488800

[B8] Perttunen K Tasmuth T Kalso E Chronic pain after thoracic surgery: a follow-up study Acta Anaesthesiologica Scandinavica 1999 43 5 563 567 10.1034/j.1399-6576.1999.430513.x 10342006

[B9] Perkins FM Kehlet H Chronic pain as an outcome of surgery. A review of predictive factors Anesthesiology 2000 93 4 1123 1133 10.1097/00000542-200010000-00038 11020770

[B10] Macrae WA Chronic post-surgical pain: 10 years on Br J Anaesth 2008 101 1 77 86 10.1093/bja/aen099 18434337

[B11] Dolin SJ Cashman JN Bland JM Effectiveness of acute postoperative pain management: I. Evidence from published data Br J Anaesth 2002 89 3 409 423 10.1093/bja/aef207 12402719

[B12] Wylde V Rooker J Halliday L Blom A Acute post-operative pain at rest after hip and knee arthroplasty: severity, sensory qualities and impact on sleep Orthopaedics and Traumatology: Surgery and Research in press 10.1016/j.otsr.2010.12.003 21388906

[B13] Beard DJ Murray DW Rees JL Price AJ Dodd CA Accelerated recovery for unicompartmental knee replacement--a feasibility study Knee 2002 9 3 221 224 10.1016/S0968-0160(02)00016-9 12126681

[B14] Capdevila X Barthelet Y Biboulet P Ryckwaert Y Rubenovitch J d'Athis F Effects of perioperative analgesic technique on the surgical outcome and duration of rehabilitation after major knee surgery Anesthesiology 1999 91 1 8 15 10.1097/00000542-199907000-00006 10422923

[B15] Jin F Chung F Multimodal analgesia for postoperative pain control J Clin Anesth 2001 13 7 524 539 10.1016/S0952-8180(01)00320-8 11704453

[B16] Adam F Chauvin M Du Manoir B Langlois M Sessler DI Fletcher D Small-dose ketamine infusion improves postoperative analgesia and rehabilitation after total knee arthroplasty Anesth Analg 2005 100 2 475 480 10.1213/01.ANE.0000142117.82241.DC 15673878 PMC1388093

[B17] Wilmore DW Kehlet H Management of patients in fast track surgery Bmj 2001 322 7284 473 476 10.1136/bmj.322.7284.473 11222424 PMC1119685

[B18] Liu SS Richman JM Thirlby RC Wu CL Efficacy of continuous wound catheters delivering local anesthetic for postoperative analgesia: a quantitative and qualitative systematic review of randomized controlled trials J Am Coll Surg 2006 203 6 914 932 10.1016/j.jamcollsurg.2006.08.007 17116561

[B19] Fu P Wu Y Wu H Li X Qian Q Zhu Y Efficacy of intra-articular cocktail analgesic injection in total knee arthroplasty - a randomized controlled trial Knee 2009 16 4 280 284 10.1016/j.knee.2008.12.012 19299145

[B20] Bianconi M Ferraro L Traina GC Zanoli G Antonelli T Guberti A Ricci R Massari L Pharmacokinetics and efficacy of ropivacaine continuous wound instillation after joint replacement surgery Br J Anaesth 2003 91 6 830 835 10.1093/bja/aeg277 14633754

[B21] Toftdahl K Nikolajsen L Haraldsted V Madsen F Tonnesen EK Soballe K Comparison of peri- and intraarticular analgesia with femoral nerve block after total knee arthroplasty: a randomized clinical trial Acta orthopaedica 2007 78 2 172 179 10.1080/17453670710013645 17464603

[B22] Andersen KV Pfeiffer-Jensen M Haraldsted V Soballe K Reduced hospital stay and narcotic consumption, and improved mobilization with local and intraarticular infiltration after hip arthroplasty: a randomized clinical trial of an intraarticular technique versus epidural infusion in 80 patients Acta orthopaedica 2007 78 2 180 186 10.1080/17453670710013654 17464604

[B23] Andersen LJ Poulsen T Krogh B Nielsen T Postoperative analgesia in total hip arthroplasty: a randomized double-blinded, placebo-controlled study on peroperative and postoperative ropivacaine, ketorolac, and adrenaline wound infiltration Acta orthopaedica 2007 78 2 187 192 10.1080/17453670710013663 17464605

[B24] Vendittoli PA Makinen P Drolet P Lavigne M Fallaha M Guertin MC Varin F A multimodal analgesia protocol for total knee arthroplasty. A andomized, controlled study J Bone Joint Surg Am 2006 88 2 282 289 10.2106/JBJS.E.00173 16452738

[B25] Busch CA Shore BJ Bhandari R Ganapathy S MacDonald SJ Bourne RB Rorabeck CH McCalden RW Efficacy of periarticular multimodal drug injection in total knee arthroplasty. A randomized trial The Journal of bone and joint surgery 2006 88 5 959 963 10.2106/JBJS.E.00344 16651569

[B26] Gomez-Cardero P Rodriguez-Merchan EC Postoperative analgesia in TKA: ropivacaine continuous intraarticular infusion Clin Orthop Relat Res 2010 468 5 1242 1247 10.1007/s11999-009-1202-2 20049572 PMC2853675

[B27] Essving P Axelsson K Kjellberg J Wallgren O Gupta A Lundin A Reduced morphine consumption and pain intensity with local infiltration analgesia (LIA) following total knee arthroplasty Acta orthopaedica 2010 81 3 354 360 10.3109/17453674.2010.487241 20450425 PMC2876839

[B28] Chen DW Hsieh PH Huang KC Hu CC Chang YH Lee MS Continuous intra-articular infusion of bupivacaine for post-operative pain relief after total hip arthroplasty: a randomized, placebo-controlled, double-blind study European journal of pain (London, England) 2010 14 5 529 534 10.1016/j.ejpain.2009.08.008 19781964

[B29] Andersen LO Husted H Otte KS Kristensen BB Kehlet H High-volume infiltration analgesia in total knee arthroplasty: a randomized, doubleblind, placebo-controlled trial Acta Anaesthesiol Scand 2008 52 10 1331 1335 10.1111/j.1399-6576.2008.01777.x 19025523

[B30] Parvataneni HK Shah VP Howard H Cole N Ranawat AS Ranawat CS Controlling pain after total hip and knee arthroplasty using a multimodal protocol with local periarticular injections: a prospective randomized study The Journal of arthroplasty 2007 22 6 Suppl 2 33 38 10.1016/j.arth.2007.03.034 17823012

[B31] Zhang J Jiang Y Shao JJ Shen H Wang Q Zhang XL Effect of periarticular multimodal drug injection on pain after total knee arthroplasty. [Chinese] Journal of Clinical Rehabilitative Tissue Engineering Research 2007 11 43 8678 8682

[B32] Essving P Axelsson K Kjellberg J Wallgren O Gupta A Lundin A Reduced hospital stay, morphine consumption, and pain intensity with local infiltration analgesia after unicompartmental knee arthroplasty Acta orthopaedica 2009 80 2 213 219 10.3109/17453670902930008 19404806 PMC2823175

[B33] Vendittoli PA Makinen P Drolet P Lavigne M Fallaha M Guertin MC Varin F A multimodal analgesia protocol for total knee arthroplasty. A randomized, controlled study The Journal of bone and joint surgery American 2006 88 2 282 289 10.2106/JBJS.E.00173 16452738

[B34] Bellamy N Buchanan WW Goldsmith CH Campbell J Stitt LW Validation study of WOMAC: a health status instrument for measuring clinically important patient relevant outcomes to antirheumatic drug therapy in patients with osteoarthritis of the hip or knee J Rheumatol 1988 15 12 1833 1840 3068365

[B35] Bellamy N WOMAC: a 20-year experiential review of a patient-centered self-reported health status questionnaire J Rheumatol 2002 29 12 2473 2476 12465137

[B36] Hawker GA Davis AM French MR Cibere J Jordan JM March L Suarez-Almazor M Katz JN Dieppe P Development and preliminary psychometric testing of a new OA pain measure - an OARSI/OMERACT initiative Osteoarthritis and Cartilage 2008 16 4 409 414 10.1016/j.joca.2007.12.015 18381179 PMC3268063

[B37] Freynhagen R Baron R Gockel U Tolle TR painDETECT: a new screening questionnaire to identify neuropathic components in patients with back pain Curr Med Res Opin 2006 22 10 1911 1920 10.1185/030079906X132488 17022849

[B38] Williams A Kind P The present state of play about QALYs Measure of the quality of life: the uses to which they may be put 1992 Hopkins A: RCP publications

[B39] Zigmond AS Snaith RP The hospital anxiety and depression scale Acta Psychiatr Scand 1983 67 6 361 370 10.1111/j.1600-0447.1983.tb09716.x 6880820

[B40] Nicholas M Self-efficacy and chronic pain Paper presented at the annual conference of the British Psychological Society: 1989; St Andrews, Scotland 1989

[B41] Moss-Morris R The Revised Illness Perception Questionnaire (IPQ-R) Psychology and Health 2002 17 1 1 16 10.1080/08870440290001494

[B42] Carver CS You want to measure coping but your protocol's too long: consider the brief COPE Int J Behav Med 1997 4 1 92 100 10.1207/s15327558ijbm0401_6 16250744

[B43] Glaser B Strauss A The discovery of grounded theory 1967 Chicago: Aldine

[B44] Tubach F Ravaud P Baron G Falissard B Logeart I Bellamy N Bombardier C Felson D Hochberg M van der Heijde D Evaluation of clinically relevant changes in patient reported outcomes in knee and hip osteoarthritis: the minimal clinically important improvement Annals of the rheumatic diseases 2005 64 1 29 33 10.1136/ard.2004.022905 15208174 PMC1755212

[B45] Schulz KF Altman DG Moher D CONSORT 2010 statement: updated guidelines for reporting parallel group randomised trials BMJ 2010 340 c332 10.1136/bmj.c332 20332509 PMC2844940

[B46] Jeffrey A Wylde V Blom AW Horwood J 'Its there an I'm stuck with it': patients' experiences of chronic pain following total knee replacement surgery Arthritis Care Res in press 10.1002/acr.20360 20890979

[B47] Kurtz S Ong K Lau E Mowat F Halpern M Projections of Primary and Revision Hip and Knee Arthroplasty in the United States from 2005 to 2030 The Journal of bone and joint surgery 2007 89 4 780 785 10.2106/JBJS.F.00222 17403800

[B48] Bhandari M Richards RR Sprague S Schemitsch EH The quality of reporting of randomized trials in the Journal of Bone and Joint Surgery from 1988 through 2000 The Journal of bone and joint surgery 2002 84-A 3 388 396 11886908 10.2106/00004623-200203000-00009

[B49] Poolman RW Struijs PA Krips R Sierevelt IN Marti RK Farrokhyar F Bhandari M Reporting of outcomes in orthopaedic randomized trials: does blinding of outcome assessors matter? The Journal of bone and joint surgery 2007 89 3 550 558 10.2106/JBJS.F.00683 17332104

[B50] Freedman KB Back S Bernstein J Sample size and statistical power of randomised, controlled trials in orthopaedics The Journal of bone and joint surgery 2001 83 3 397 402 10.1302/0301-620X.83B3.10582 11341427

[B51] Chan S Bhandari M The quality of reporting of orthopaedic randomized trials with use of a checklist for nonpharmacological therapies The Journal of bone and joint surgery 2007 89 9 1970 1978 10.2106/JBJS.F.01591 17768194

